# Ultra-High Sensitivity and Temperature-Insensitive Optical Fiber Strain Sensor Based on Dual Air Cavities

**DOI:** 10.3390/ma16083165

**Published:** 2023-04-17

**Authors:** Zhiqi Lu, Changning Liu, Chi Li, Jie Ren, Lun Yang

**Affiliations:** 1 College of Physics and Electronic Science, Hubei Normal University, Huangshi 435002, China; 2 Institute for Advanced Materials, Hubei Normal University, Huangshi 435002, China; 3 Laboratory of Solid-State Microstructures, Nanjing University, Nanjing 210093, China

**Keywords:** femtosecond laser machining, strain sensor, interferometers, microcavities

## Abstract

This study proposed an all-fiber Fabry–Perot interferometer (FPI) strain sensor with two miniature bubble cavities. The device was fabricated by writing two axial, mutually close short-line structures via femtosecond laser pulse illumination to induce a refractive index modified area in the core of a single-mode fiber (SMF). Subsequently, the gap between the two short lines was discharged with a fusion splicer, resulting in the formation of two adjacent bubbles simultaneously in a standard SMF. When measured directly, the strain sensitivity of dual air cavities is 2.4 pm/με, the same as that of a single bubble. The measurement range for a single bubble is 802.14 µε, while the measurement range for a double bubble is 1734.15 µε. Analysis of the envelope shows that the device possesses a strain sensitivity of up to 32.3 pm/με, which is 13.5 times higher than that of a single air cavity. Moreover, with a maximum temperature sensitivity of only 0.91 pm/°C, the temperature cross sensitivity could be neglected. As the device is based on the internal structure inside the optical fiber, its robustness could be guarantee. The device is simple to prepare, highly sensitive, and has wide application prospects in the field of strain measurement.

## 1. Introduction

Fiber optic sensors have been employed in a wide range of applications, including the measurement of several physical quantities such as pressure [1,2,3], salt content [4,5], vibration [6,7,8], refractive index [9,10,11], temperature [12,13,14], strain [15,16], micro-displacement [17], and relative humidity [18]. Owing to their advantages, such as compact size, light weight, immunity to electromagnetic interference, high sensitivity, wide temperature range, stability, and durability, optical fiber sensors play a pivotal role in the industry. In particular, strain measurement is an extensively used fiber optic sensing technology because of its ability to perform medical monitoring and civil engineering. Recently, Mach–Zehnder interferometers [19,20] (MZIs), long-period gratings [21] (LPGs), and particularly fiber Bragg gratings [22,23] (FBGs), have garnered attention in the field of strain-sensing technology; however, none of them exhibit low temperature sensitivity, which increases the cross-sensitivity between temperature and strain. Consequently, temperature compensation devices inevitably increase the cost and complicate the overall system. In contrast, strain sensors based on fiber optic Fabry–Perot interferometers (FPIs) can obtain extremely low temperature cross-sensitivity, as tiny air cavities created in the fiber exhibit a small thermal expansion coefficient, where splicing techniques are used to fabricate fiber optic FPI sensors. Certain methods employed are hollow-core photonic crystal fiber splicing with single-mode fiber [24,25], capillary splicing with single-mode fiber [26,27], and two single-mode fibers dipped in liquid for splicing [28]. Owing to the large Young’s modulus of optical fiber materials, the strain sensitivity of these sensors can be limited to typically only a few pm/µε. In general, the strain sensitivity of fiber optic sensors is increased at the expense of its robustness, for instance, tapered fibers or microfibers [29,30], which have a relatively small measurement range. Therefore, the investigation of sensors that are simultaneously insensitive to temperature and can increase strain sensitivity while not reducing the measurement range are of significance. Furthermore, whether the splicing process can be omitted remains a question worth exploring.

To simultaneously measure multiple parameters or to enhance measurement sensitivity, fiber optic sensors with a dual-cavity structure are commonly used. A dual-cavity fiber Fabry–Pérot interferometer (DCFFPI) has been proposed for the measurement of relative humidity and temperature [31]. Lee et al. proposed a novel configuration based on a DCFFPI that can simultaneously measure the thermo-optic coefficient (TOC) and thermal expansion coefficient (TEC) of a polymer [32]. Pullteap et al. proposed a method for automated fringe counting of a dual-cavity extrinsic fiber Fabry–Perot interferometer for vibration measurements [33]. A DCFFPI sensor has also been used to measure both temperature and pressure [34,35]. These sensors cascade a fiber-optic intrinsic Fabry–Perot interferometer (IFPI) and an extrinsic Fabry–Perot interferometer (EFPI). These two cavities were used to measure their different parameters.

This study proposed and demonstrated a novel all-fiber FPI strain sensor with two miniature bubble cavities fabricated without splicing. To the best of our knowledge, this is the first study to report the simultaneous increase in the strain sensitivity and measurement range. Moreover, as the temperature sensitivity of the proposed sensor was only 0.91 pm/°C, the temperature cross-sensitivity could be neglected. Thus, no temperature compensation was needed, which rendered the entire measurement system simple in nature. This device was fabricated by first writing two axial, mutually close short-line structures in the core of a single-mode fiber (SMF) by femtosecond laser direct writing. Subsequently, the gap between the two short lines was discharged using a fusion splicer, which concurrently produced two bubbles close to each other in a SMF. When measured directly, the strain sensitivity of dual air cavities was 2.4 pm/με (same as that of one bubble); however, the measurement range was doubled. Further, analysis of the envelope indicates that the device exhibits a strain sensitivity of up to 32.3 pm/με, which is 13.5 times higher than that of a single air cavity. The prepared device can be used as a promising strain sensor with desirable high sensitivity strain, a relatively large measurement range, and negligible temperature sensitivity.

## 2. Device Fabrication

**Figure 1:**
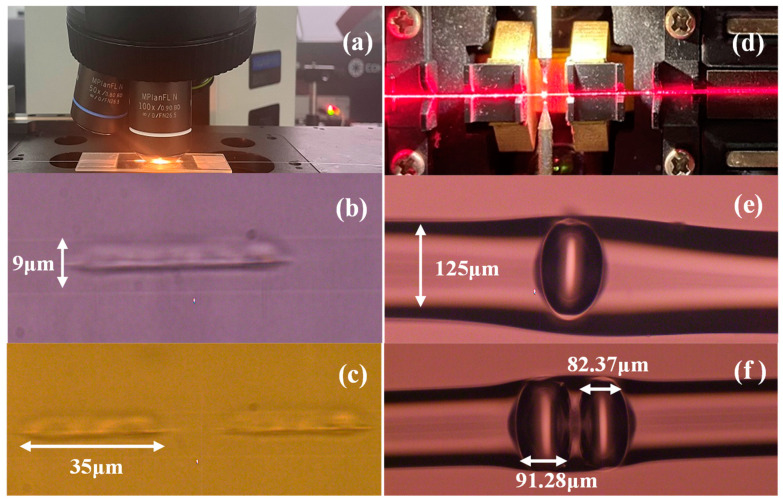
(**a**) Fabrication system for femtosecond laser scribing; (**b**) a horizontal short-line structure inscribed in fiber core; (**c**) two horizontal short-line structures inscribed in fiber core; (**d**) discharge the short-line area; (**e**,**f**) one or two bubbles are formed.

Figure 1 shows the physical flow chart of the all-fiber FPI strain sensor fabrication system. A chirped pulse amplification laser system (Coherent Inc., Santa Clara, CA, USA) delivered 35 fs laser pulses with a repetition rate of 1 kHz at a central wavelength of 800 nm. The pulses were focused on the fiber core through an objective lens (100×, Olympus Corp., Shinjuku City, Tokyo) with a numerical aperture (NA) of 0.9. The femtosecond laser pulse energy was approximately 1.2 µJ, which was adjusted using a variable-density filter. The SMF (9 µm/125 µm, YOFC, Wuhan, China) was held horizontally by the clamp on a laser micromachining platform (Prior Scientific Instruments Ltd., Cambridge, UK), which exhibited a three-dimensional motion controlled by a computer, a resolution of 1 μm, as shown in Figure 1a.

There are two steps in the fabrication process:

1. As illustrated in Figure 1b, a 35 µm short-line structure is inscribed on the fiber core using a femtosecond laser pulse at a scanning speed of 10 µm/s. Each transverse short-line structure is written in just 3.5 s. The same procedure is used to inscribe two short-line structures on the fiber, each 35 µm long and 15 µm apart, as shown in Figure 1c. This process does not create a cavity; it merely modifies the fiber’s refractive index;
2. The area where the refractive index has been altered by direct writing with the femtosecond laser is observed using a laser pass-through pen. When the laser travels through the laser-etched zone, it experiences a relatively high insertion loss, producing a bright spot when the laser passes through it, as shown in Figure 1d. The fiber with only one horizontal short structure is placed in a fusion splicer and discharged against the splicer’s center to form an air cavity 95.35 µm wide, as demonstrated in Figure 1e. The first fiber optic sensor containing a single bubble was fabricated. Subsequently, the second fiber sensor with a double bubble is created by discharging a fiber fusion machine in the gap between two short structures, resulting in two air cavities close to each other in a standard SMF with widths of 82.37 µm and 91.28 µm and a spacing of about 21 µm between them, as shown in Figure 1f. Next, we compare the performance of single-bubble and double-bubble fiber sensors.

The device’s sensing length is less than 200 µm, indicating a particularly compact structure. A JILONG KL-300T fusion splicer was used, and the fusing current and duration were 10 mA and 1.5 s, respectively. It is important to note that once the femtosecond laser energy, the discharge current, and time of the fiber fusion machine have been determined, the microbubble parameters remain stable and reproducible. The microcavity is approximately 90 µm in size, and minor variations in its width have little effect on the strain sensitivity of the fiber optic sensor [28].

## 3. Operation Principle of the Device

The incident beam travels along the core of the SMF, and is reflected on both surfaces of the FP cavity, eventually recombining in the core to form interference fringes at the output. The intensity of the reflected beam at the two interfaces of a single FP cavity was set to *I*_1_ and *I*_2_, respectively, and the interference intensity could be expressed as follows.

(1) $$I=I_{1}+I_{2}+2\sqrt[{}]{I_{1}I_{2}}\cos\;(\frac{4\pi n_{air}L}{\lambda }+\phi_{0})$$

where 𝜆 is the wavelength of the propagation light, *n*_air_ is the effective refractive index of the microcavity, *L* is the cavity length of the FPI, and 𝜙_0_ is the initial phase of the interference. The normalized signals intensity from individual FPI within the fiber can be simplified expression as follows:

(2) $$I\;(L)=\cos\;(\frac{4\pi n_{air}L}{\lambda })$$

when the condition 4π*n_air_L*/*λ*_m_ = (2*m* + 1)π is satisfied, where *𝑚* is an integer and the specific resonance peak appears at the wavelength

(3) $$\lambda_{m}=\frac{4n_{air}L}{2m+1}$$

If the sensing structure containing bubbles is attached to the platform ends and axial strain is applied, the bubble microcavity length increases. The axial strain is expressed by *ε*.

(4) $$\varepsilon =\;\frac{F}{S\times E}$$

Young’s modulus (*E*) for fused silica is 70.3 GPa. *F* is the force applied and *S* is the cross-sectional area. As *F* increases, axial strain (*ε*) and bubble microcavity length increase, causing interference spectrum drift.

The microbubble can be regarded as an ellipsoid. It is possible to approximate the change in axial length (Δ*l*) of its microcavity under the influence of axial force.

(5) $$\Delta l\;=\;2\;\int_{0}^{a}\;\frac{F}{S\text{·}E}\;\;\mathrm{dx}=\;\frac{2F}{E}\;\int_{0}^{a}\;\frac{1}{S}\;\;\mathrm{dx}=\frac{2F}{E}\beta$$

The initial axial length of the bubble cavity is 2*a*, *β* is determined by the elliptic parameter. We can conclude from Equation (5) that the strain in the bubble cavity is linearly related to the applied force, and from Equation (3) that the wavelength drift of the resonant peak is also linearly related to the strain.

The free spectra range (*FSR*) of the interference fringe of the FP microcavity can be given by

(6) $$FSR_{s}=\frac{\lambda^{2}}{2nL_{s}},\;FSR_{r}=\frac{\lambda^{2}}{2nL_{r}}$$

where *L*_s_ and *L*_r_ are the cavity lengths of the sensing and reference FPI, respectively. The output light intensity of a sensor comprising two FPIs cascaded by a sensing cavity and a reference cavity can be expressed as

(7) $$I(L_{s},L_{r})=\cos[\frac{2\pi n_{air}(L_{s}+L_{r})}{\lambda }]\cos[\frac{2\pi n_{air}(L_{s}-L_{r})}{\lambda }]$$

The high frequency component of the output signal oscillates rapidly and the low frequency component generates an envelope. The *FSR_envelope_* could be described as [36,37]

(8) $$FSR_{envelope}=\;\;\frac{FSR_{s}FSR_{r}}{\left|FSR_{s}-FSR_{r}\right|}$$

Thus, the wavelength shift of the dual-cavity envelope produces an amplification compared to a single sensing microcavity. The two cavity lengths determine exactly the amplification factor, which can be expressed as [38].

(9) $$M=\;\;\frac{FSR_{envelope}}{FSR_{s}}\;=\;\;\frac{FSR_{r}}{\left|FSR_{s}-FSR_{r}\right|}=\;\frac{L_{s}}{\left|L_{r}-L_{s}\right|}$$

It can be easily seen that the magnification depends only on the cavity lengths of the two cavities, and a vernier effect occurs when their lengths are close but not equal. We can obtain a periodic reflection spectrum, which consists of a series of stripes with different amplitudes. Theoretically, the closer the difference in cavity lengths between the sensing and reference cavities, the larger the amplification factor. However, when the difference between the cavity lengths of the two cavities is too small, the *FSR* of the envelope becomes too large and the envelope point is outside the wavelength range of the broadband light source, thus, creating adversity in the course of the experiment.

Wang et al. [39] prepared a 75 μm long FPI cavity and obtained a temperature sensitivity of 0.273 pm/°C near 1550 nm, which was negligible. This means that when the ambient temperature changes, it does not affect the strain measurement of the FPI cavity.

**Figure 2:**
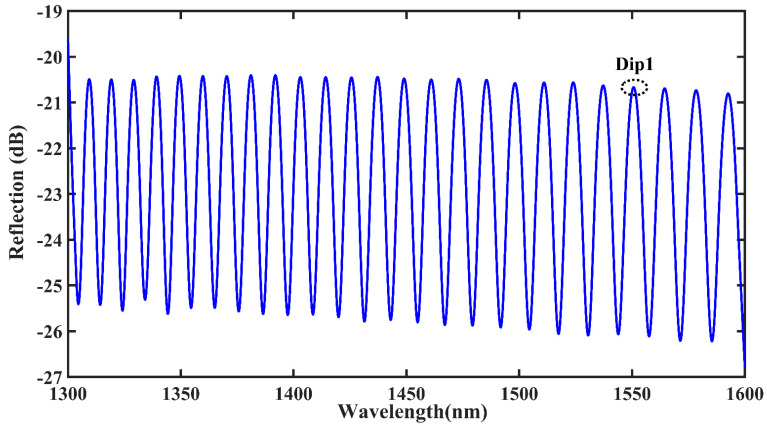
Original spectrum of single bubble.

**Figure 3:**
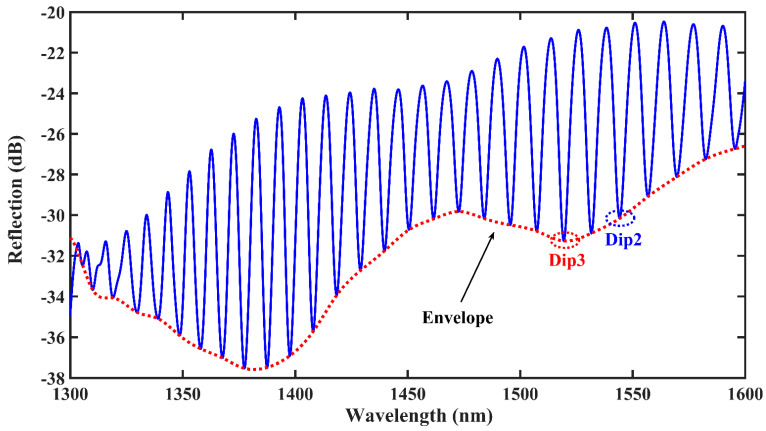
Original spectrum of double bubbles.

The initial spectrum of a single bubble is depicted in Figure 2, and dip1 at approximately 1550 nm in the communication band is considered as the object of study. The initial spectrum of the double bubble is illustrated in Figure 3, and dip2 at approximately 1550 nm in the communication band is considered as the next object of study. As the double bubbles are closer in size, they interact with each other and the initial spectrum exhibits a clear downward envelope, as shown in the red curve in Figure 3. Further, dip3 at roughly 1520 nm is considered as the third object of study.

## 4. Experimental Results and Discussion

### 4.1. Axial Strain Experiment

**Figure 4:**
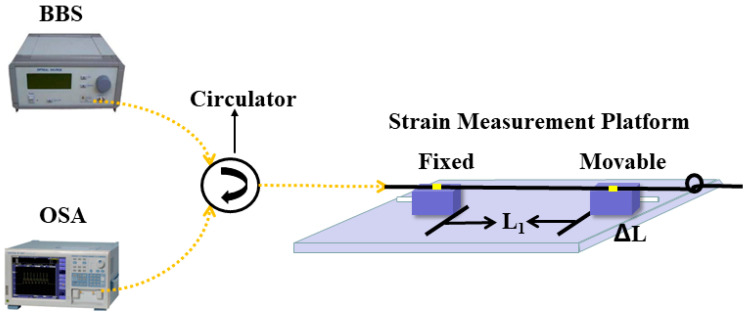
Schematic of strain experimental setup.

First, The Broadband Light Source (BBS, FL-ASE) and Optical Spectrum Analyzer (OSA, YOKOGAWA, AQ6370D) were connected to the SMF at both ends of the sensor. The spectral range of BBS adopted in the experiments was 1250–1650 nm, the maximum measurement range of OSA was 600–1700 nm with an accuracy of ±0.02 nm. Then, the fabricated sensing structures were placed in setups as in Figure 4 for axial strain experiments.

As shown in Figure 4, the sensor structure was fixed on two horizontal and equal height precision displacement platforms with UV glue, and the experiment was started after the glue was completely dried. To begin with, the displacement platform was adjusted so that the sensor was in a straightened state. Subsequently, the micrometer of the precision displacement platform on the right side was adjusted, where one frame of micrometer was adjusted each time and a set of data was recorded. Axial strain calculation formula is

(10) ε=Δdd

where Δ*d* is the total movement of the displacement stage, *d* is the distance between two fixed points in the initial state, and *ε* is the axial strain. The distances between the two fixed points of the single-bubble and double-bubble sensing structures were 18.7 cm and 17.3 cm, respectively. The right knob was adjusted one frame at a time, which was 10 μm. Moreover, the data were recorded after the spectrum was stabilized to ensure the accuracy of the experiment.

**Figure 5:**
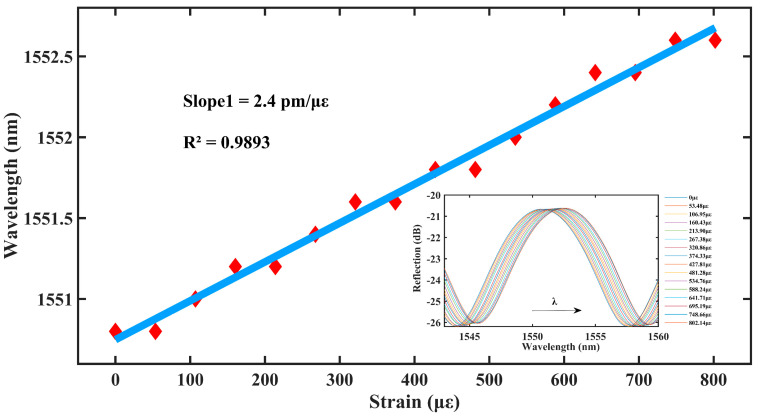
Single bubble of strain drift and fit plots.

As shown in Figure 5, the central wavelength of the sensor resonance peak dip1 is red-shifted within the axial strain range of 0–802.14 με. The sensitivity of the sensor to axial strain reaches 2.4 pm/με, and the linearity fit reaches 0.9893, thus, exhibiting an excellent linear relationship and high sensitivity.

**Figure 6:**
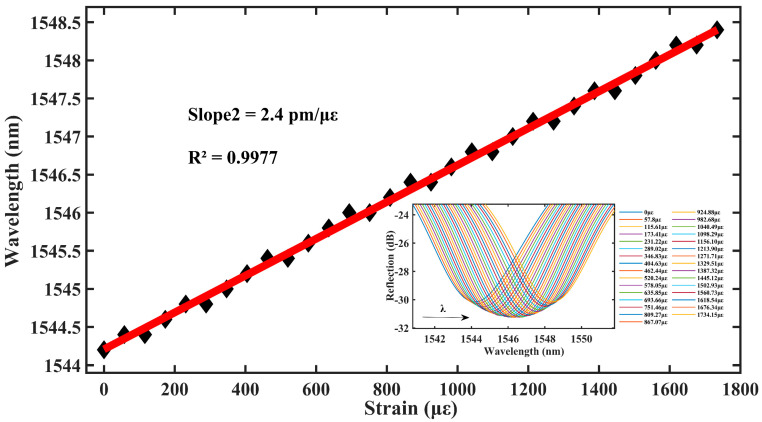
Double bubble strain drift and fit plots before magnification.

As shown in Figure 6, the central wavelength of the sensor resonance peak dip2 is red-shifted with increase in the axial strain intensity. In the axial strain range of 0–1734.15 με, the sensitivity of the axial strain of the fiber sensor remains 2.4 pm/με with a linear fit of 0.9977. This indicates that the range of the double bubble is more than double compared with that of the single bubble, although the sensitivity of the strain remains the same.

**Figure 7:**
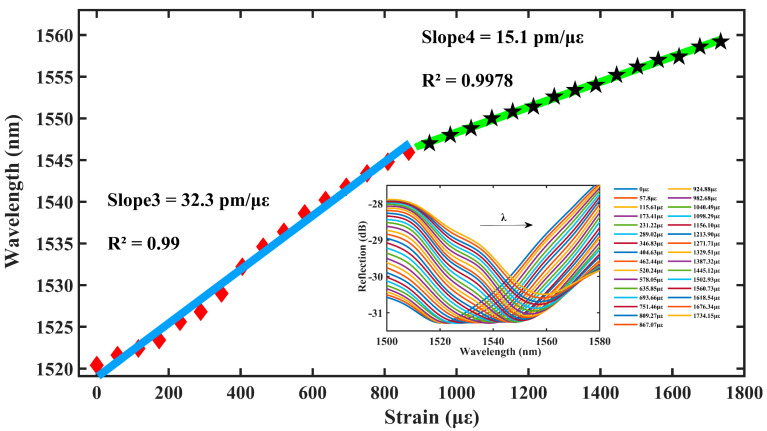
Double bubble strain drift and fit plots after magnification.

As shown in Figure 7, the red envelope of Figure 3 is used as a drift plot, and the central wavelength of the sensor resonance peak dip3 is continuously red-shifted when the intensity of the axial strain increases. In the axial strain range of 0–867.07 με, the strain sensitivity of the sensor is 32.3 pm/με with a linear fit of 0.99; in the axial strain range from 867.07–1734.15 με, the strain sensitivity of the sensor is 15.1 pm/με with a linear fit of 0.9978.

It is assumed that one of the microcavities is not affected by the external environment and is considered as the reference cavity, while the other is the sensing cavity. Theoretically, we substitute the lengths of the two microcavities into Equation (9) to find the magnification of 9.24 or 10.24.

The amplification effect is further complicated by the fact that both microcavities are subjected to strain at the same time in this experiment. The results show that the two microcavities respond to strain in opposite directions in the first half range of 0–867.07 με, thus, enhancing the vernier effect on sensitivity with a magnification of 13.45. In the second half range of 867.07–1734.15 με, the response to the strain is in the same direction, thus, weakening the effect of the vernier effect on the sensitivity with a magnification of 6.29. Moreover, the wide strain measurement ranges up to 1734.15 με, which indicates the robustness of the device.

### 4.2. Temperature Experiment

To investigate the stain sensitivity of the fiber optic sensor, the cross-sensitivity to temperature must be considered.

**Figure 8:**
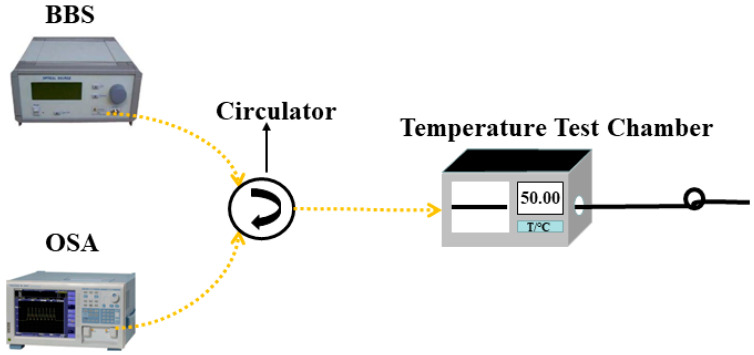
Schematic of temperature experimental setup.

The experimental setup for sensor temperature measurement is shown in Figure 8. The fabricated sensor structure was straightened and placed horizontally in the middle of the experimental chamber (OTF-1200X, KEJING Materials Co., Hefei, China) with one end connected to BBS and OSA, and the other end was cut diagonally to avoid reflections from the end face. A programmable constant temperature test chamber was used in the experiment to simulate the temperature change of the external environment with a temperature accuracy of ±0.1 °C. The temperature experiment was ramped up uniformly from 25–80 °C, and the reflection spectra of the sensors were recorded at an interval of 5 °C.

**Figure 9:**
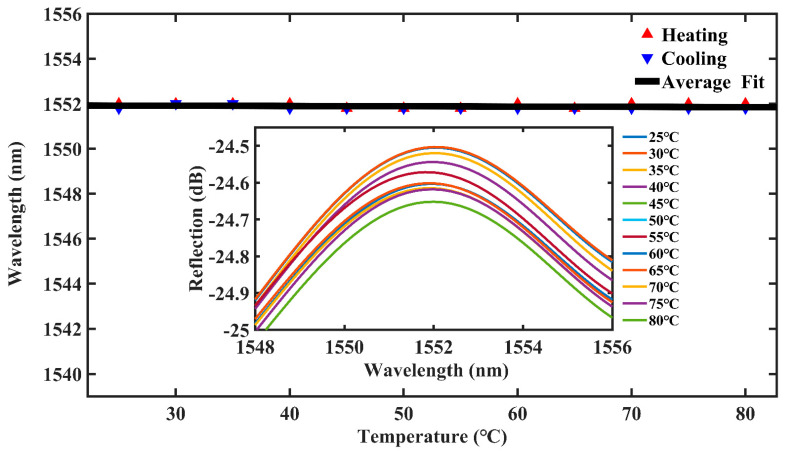
Experiment setup of the temperature sensing system based on cascaded FPIs.

To ensure the repeatability of the sensor, we conducted a temperature reduction experiment in steps of 5 °C from 80 °C to 25 °C. By analyzing the data, we find that the structure’s sensitivity to temperature is also very weak. As shown in Figure 9, it can be seen that the temperature changes from 25 °C to 80 °C have a small effect on the intensity of the spectra. A comprehensive observation of the temperature rise and fall shows that the maximum drift is only 0.05 nm. Therefore, the maximum sensitivity to temperature fluctuations of 0.91 pm/°C can be seen to be almost negligible. The cross effect of temperature could be neglected, additional temperature compensation was no longer needed, and the sensing system could be greatly simplified.

Table 1 gives a comparison between our sensor and previously reported sensors, and our sensor performs very well in terms of a comprehensive evaluation of measurement range, strain sensitivity, and temperature cross-sensitivity.

## 5. Conclusions

In summary, this study proposed a new type of FPI strain sensor with dual air cavities in the SMF, which was fabricated via femtosecond laser direct writing of two short lines in the core of a standard SMF, followed by discharge using a fiber fusion splicer. Owing to the high temperature generated by the fiber fusion, the line structure instantly expands and forms two separate microcavities simultaneously. The strain sensitivity is increased to 32.3 pm/με with a measuring range of 1734.15 µε. The strain sensitivity has a maximum magnification of 13.45 and the cross-sensitivity of temperature is very small and negligible. Thus, the interferometer allows the design of temperature-independent and ultrasensitive strain sensors. The prepared device is compact, with the sensing area being less than 200 μm in length, and it is easy to fabricate. Furthermore, the proposed fiber optic FPI strain sensor offers the advantages of robust structure and stable measuring, all of which make it attractive for many micro-strain sensing fields.

## Figures and Tables

**Table 1:** Comparisons of the proposed sensors with other representative structures.

Ref.	Device Structure	Strain Sensitivity	Measurement Range of Strain	Temperature Sensitivity
[22]	FBGs	1.21 pm/µε	0–3138 με	14.91 pm/°C
[24]	Spheroidal FP cavities	10.3 pm/με	0–1100 με	1 pm/°C
[25]	Hollow-core PCF	1.89 pm/µε	0–4000 με	5.58 pm/°C
[26]	Cascaded FP cavities	2.97 pm/με	0–1000 με	0.9 pm/°C
[27]	FPI with air cavity	3.29 pm/με	0–1100 με	1.08 pm/°C
[28]	FPIs	6.0 pm/με	0–1000 με	1 pm/°C
[29]	Tapered-based TCF	6.11 pm/με	0–841.5 με	0.69 pm/°C
[30]	S-tapered with MMF	103.8 pm/με	0–170 με	36.2 pm/°C
[38]	FPI based on Vernier effect	28.11 pm/με	0–1500 με	278.48 pm/°C
This work	FPIs	32.3 pm/με	0–1734.15 με	0.91 pm/°C

## Data Availability

Data underlying the results presented in this paper are not publicly available at this time but may be obtained from the authors upon reasonable request.
